# What are the outcomes of marine site protection on poverty of coastal communities in Southeast Asia? A systematic review protocol

**DOI:** 10.1186/s13750-022-00255-1

**Published:** 2022-02-04

**Authors:** Mohd Aizat Zain, Julia Suhaimi, Maznah Dahlui, Hong Ching Goh, Amy Yee-Hui Then, Nur Asyikin Yakub, Mohd Iqbal Mohd Noor, Ruth Garside, Jacqualyn Eales, Edgar Jose, Fatimah Kari

**Affiliations:** 1 Department of Urban and Regional Planning, Faculty of Built Environment, Universiti Malaya, Kuala Lumpur, Malaysia; 2 Department of Basic Health Sciences, Faculty of Pharmacy, MAHSA University, Kuala Lumpur, Malaysia; 3 Department of Primary Care Medicine, Faculty of Medicine, Universiti Malaya, Kuala Lumpur, Malaysia; 4 Department of Social and Preventive Medicine, Faculty of Medicine, Center for Population Health, Universiti Malaya, 50603 Kuala Lumpur, Malaysia; 5 Faculty of Public Health, Universitas Airlangga, Surabaya, Indonesia; 6 Faculty of Science, Institute of Biological Sciences, Universiti Malaya, Kuala Lumpur, Malaysia; 7 Faculty of Business Management, Universiti Teknologi MARA, 26400 Shah Alam, Pahang Malaysia; 8 European Centre for Environment and Human Health, College of Medicine and Health, University of Exeter, Knowledge Spa, Truro, TR1 3HD UK; 9 College of Fisheries and Aquatic Sciences, Western Philippines University, Puerto Princesa, Palawan Philippines; 10 Centre for Civilisational Dialogue, Universiti Malaya, Kuala Lumpur, Malaysia

**Keywords:** Sea, Human population, Economic, Conservation, Living standard, Well-being, Marine protected area (MPA)

## Abstract

**Background:**

Many conservation management interventions have been set up to bring win–win outcomes for both biodiversity conservation and the well-being of the local communities. Nevertheless, the implementation process of marine protected areas (MPAs) can generate unexpected outcomes and fail to reach its objectives in addressing communities’ challenges. Therefore, it is crucial to have a better understanding of how MPAs influence the socioeconomic aspects of the coastal communities. This paper describes the protocol to conduct a systematic review which aims to explore and review the evidence that reflects the outcomes of marine site protection on poverty reduction in terms of economic and material living standards among the coastal communities in Southeast Asia. The review question is “What are the outcomes of marine site protection implementation on poverty in terms of material and economic living standards of coastal communities in Southeast Asia?”.

**Methods:**

The systematic review uses rigorous search strategies and selection methods based on pre-defined eligibility criteria to identify and examine published journal articles and grey literature that are available on the review topic. Relevant studies and grey literature will be extracted from a recent systematic map of the evidence documenting the effect of marine or coastal nature conservation or natural resource management activities on human well-being in Southeast Asia. We will search online databases including Web of Science Core Collection, Ovid Medline^®^, Environmental Complete, Scopus, as well as Google Scholar and sources of grey literature for any additional literature available since the evidence map was created. For this review, the populations of interest are from coastal communities in the Southeast Asia region. Comparators to marine site protection will be no intervention and/or pre-MPA implementation. The economic and material living standards, which are the poverty domains, will be evaluated as outcomes. Once we have identified relevant literature, we will perform a critical appraisal, data extraction, and synthesis appropriate to the type of literature found, to investigate the effect of marine site protection on poverty reduction.

**Supplementary Information:**

The online version contains supplementary material available at 10.1186/s13750-022-00255-1.

## Background

In the early 1980s, there was a paradigm shift in conservation from the prevailing top-down protectionist approach to an approach that is more sensitive to the rights and needs of local people [1]. The shift occurred partly due to the disproportionate costs of conservation imposed on often poorer communities in developing countries, and due to the recognition of importance of gaining local communities’ support to achieve conservation goals [2, 3]. This led to the introduction of the concept of integrated protected areas, which has dual goals of nature conservation and poverty reduction among the local community. Despite the goals having been reinvented this way, the evaluation in terms of social outcome of many protected areas remains poorly understood due to the lack of evaluation in this area as compared to that in the biological domains. In addition, there has been mixed evidence of the direction and strength of reported social outcomes of protected areas [2, 4, 5].

The definition of coastal community is not consistent across the literature, but it can be described as a human settlement along a thin strip of coastal land or on the water along the boundaries between the sea and the land, including seaside towns and ports [6]. Coastal communities have benefited in terms of human health and well-being from numerous ecosystem services provided by the sea and coastal areas such as protein-rich food, economic development of the tourism sector, commercial fisheries, sociocultural benefits, and natural defence against floods and storms. Demand for these ecosystem services has increased, despite the acknowledgment of the marine environment deterioration caused by it. This has led to a global increase of systematic and integrative marine governance especially in Southeast Asian (SEA) countries, which consist of many low- and middle-income countries with richness and abundance of biodiversity owing to their tropical ecosystem [7].

Several coastal communities including those in the SEA region have reported problems with the accessibility of sufficient water, food, and energy sources [8–11]. Furthermore, the implementation of marine site protection in coastal areas can be challenging for coastal communities especially for smallholder farmers, fishermen, and vulnerable and marginalised populations [2, 12–15]. The loss of economic opportunities together with a frequent lack of infrastructure in the underdeveloped areas may then undermine their health, nutrition, and quality of life [6].

On the other hand, there is also evidence for the positive effects of MPAs on the welfare of coastal communities. In a previous review article that examined the outcomes of MPA establishment on human welfare, it was concluded that food security among local people remained stable or increased following MPA establishment. An increase was also observed in resource rights of the locals, and the effects were positively correlated with the MPA zoning [16]. Moreover, a previous study showed that the local coastal communities in the United Kingdom generally perceived improvement in their social, economic, and environmental benefits after 2 years (2008–2010) of MPA establishment in Lyme Bay, United Kingdom [17]. Taken together, the outcomes of MPAs on human welfare or well-being appear mixed. The heterogeneity is perhaps due to the different geographical locations, different marine policies and governances, and the sociocultural situations of the coastal communities. Therefore, it has been assumed that if MPAs are established, it will change the economic and material living standards of coastal communities because MPAs can result in increased local fish catch but can also restrict access to marine resources, especially among marginalised and vulnerable populations.

There are various systematic review methodologies [18]. This paper aims to describe the agreed process that will be undertaken when conducting the systematic review which will gather evidence from many different countries and different authors, so that a standardised process can be followed. This systematic review will synthesise the available evidence on the outcome of marine site protection on poverty among the coastal communities in the SEA region. By systematically reviewing the evidence and context of individual case studies, this greater understanding can support efforts to minimise any negative effects of MPA on the local community [19–22]. The information may be used as evidence to formulate regulations related to conservation and human welfare in the marine site protection areas which could help increase acceptance of the local community towards MPA policy. This is important as the policymakers in the future may establish more coastal areas as MPAs based on the current trend.

### Stakeholder engagement

The systematic review topic and questions have been formulated based on the findings/outcomes from a stakeholder meeting conducted on 22 March 2018 at Kota Kinabalu, Sabah. The attendees of the stakeholder meeting included government agencies, non-governmental agencies (NGOs), tour operators, and the local communities of Sabah. The research team had conducted several interviews with key local stakeholders in Tun Mustapha Park (TMP), Sabah, including coastal communities and representatives from relevant government agencies to further gather insights and develop the framework of the study. Additional input and feedback were sought from researchers in the wider Blue Communities Programme team, developmental and fisheries experts, local and international NGOs via informal meetings, interviews, and conversations.

## Objectives of the review

The primary objective of the proposed systematic review is to examine the effect of implementation of marine site protection on poverty among the coastal communities in SEA. The definition of poverty in this study follows the definition provided by World Bank. Those who live on less than US$1.90 per day is considered as extreme poverty, whereas those who live on between $1.90 and $3.10 per day is considered as moderate poverty. In addition, we will also refer to the poverty line provided by the government of the research country. The present study is limited to the poverty measurement on economic and material living standards as defined in Table 2. These two domains were chosen because both domains are commonly reported in the site protection literature, based on the recent systematic map that focused on the SEA region [7]. We acknowledge that a multidimensional poverty concept may reflect a better assessment of poverty level in a population as compared to the income-based measurement as suggested by the World Bank. Nevertheless, it was not considered due to the difficulty in having a common standard measure of poverty across different studies in this review.

### Review question

This protocol for a systematic review is intended to answer the following question: “What are the outcomes of marine site protection implementation on poverty in terms of material and economic living standards of coastal communities in SEA?”. The components of the question according to the PICO (Population–Intervention–Comparator–Outcomes) framework are listed as follows:

Population: coastal communities in Southeast Asia (including communities living within the coastline and on islands).

Intervention: marine site protection.

Comparator: any comparator which falls under one of the followings; (i) a different site with no marine protection, (ii) the same site, before the marine protection was implemented, (iii) a different site, with another marine protection implementation.

Outcomes: economic and material living standards.

## Identification of review topic

A recent systematic map by Eales et al. [7] identified 281 studies on the outcome of marine management and conservation interventions on human well-being in the developing countries of SEA. The systematic map gives a general overview on the available database of evidence for on-site protection interventions to human health and well-being. It also identified several knowledge clusters, including studies that investigated the links between marine site protection and economic living standards. The present systematic review will use the information and literature gathered from the systematic map to identify relevant studies related to marine site protection and poverty reduction in the coastal communities from the systematic map. We will also search for additional documents from the online databases and grey literature. Like the systematic map, the present study will investigate all the evidence from 11 SEA countries.

## Methods

In the systematic review, the marine site protection will be defined as the marine sites that are either being managed formally by the ‘national and local government’, ‘non-governmental organisations’, or ‘people’s organisations’ for environmental conservation such as Marine Protected Area (MPA). The systematic review will follow the guidelines for systematic reviews from environmental management issued by the Collaboration for Environmental Evidence [23] and the guideline from Reporting Standards for Systematic Evidence Syntheses (ROSES) (Additional file 2) [24].

### Studies identified by the systematic map

Part of the developed systematic map [7] yielded a library of studies that examined the interaction between marine site protection and poverty reduction in SEA. Specifically, the map compiled 129 documents that described the interactions across the topics of site protection to economic and material living standards of coastal communities in the SEA region. These documents will be the primary materials used in this study. In this systematic map, the last bibliographic database search was undertaken in August 2019. Additional searches will be undertaken from August 2019 onwards at online databases of Web of Science Core Collection, Ovid Medline^®^, Environmental Complete, Scopus, and Google Scholar platforms to extract recently available literature. Relevant grey literature will be extracted from the systematic map, and we will perform additional grey literature searches in the websites described in Additional file 3.

We will remove duplication of all retrieved documents from our search strategies and the previous systematic map by using EndNote remove duplication feature to avoid redundancy during screening. A worksheet (Excel) will be created containing information on every step taken including information such as search date, search string used, search restrictions, source organisation, and web address.

### Search strategy

The search string was developed in two sub-strings of intervention and outcome terms. We adapted the search strings from the recent systematic map [7]. The search string in Table 1 will be used in the search of databases from the Web of Science Core Collection, Scopus, Ovid Medline^®^, and Environmental Complete. The search string will be adapted for Google Scholar according to its search capability.

**Table 1:** Search string that will be used in the search of the literature in the Web of Science Core Collection, Scopus, Ovid Medline® and Environmental Complete databases

Search string	
Intervention terms	(“marine protect*” OR “marine reserve*” OR “marine refug*” OR “marine park*” OR “partial closure” OR “no$take zone” OR “no$trawl*” OR “marine conservation zone” OR “nature park”) OR “mangrove protect*” OR “mangrove reserve*” OR “mangrove refug*” OR “mangrove park*” OR mangrove management) AND
Outcome terms	(poverty OR impoverish* OR unemploy* OR non-employ* OR depriv* OR disadvantaged OR disparit* OR underprivilege* OR inequit* OR inequalit* OR needy* OR “resource$poor” OR penury* OR privation* OR destitut* OR vulnerab* OR “socio$econom*” OR “low$income” OR economy OR economic* OR inequit* OR equit* OR “resource$rich” OR advantaged OR employ* OR “high$income” OR income OR livelihood* OR “standard of living” OR “living standard”)

## Comprehensiveness of the search

Eleven key research papers that are relevant to the MPA and economic and material living standards have been identified from the systematic map [7]. These studies (attached in Additional file 1) were used as benchmark studies when the search strings were developed. Initial scoping performed in Web of Science Core Collection, Scopus, Ovid Medline^®^, Environmental Complete, and Google Scholar with the search string resulted about 3658 potentially relevant articles.

## Article screening and study eligibility criteria

Articles will be evaluated for inclusion at two successive stages by using the study inclusion criteria described in Table 2. At Stage 1, two reviewers (R1 and R2) will screen the titles and abstracts to agree on which papers to include or exclude. Reviewers will review the abstracts and reject those not aligned with the eligibility criteria in Table 2, while documenting the decisions.

**Table 2:** Eligibility criteria

Criteria	Inclusion	Exclusion
Types of study	Any primary study with a comparator, including observational and manipulative	Simulation studies, review papers, and policy discussions
	Studies which are in the English language. Note: majority of conservation papers from previous systematic map related to SEA regions are in English, hence the language of choice	Studies which are in languages other than English
Population	Populations in the villages/urban areas within the coastline (any community classified as coastal by the authors of each study) in the SEA region. SEA is composed of 11 countries: Brunei, Burma (Myanmar), Cambodia, Timor-Leste, Indonesia, Laos, Malaysia, Philippines, Singapore, Thailand, Vietnam	Populations outside of the coastline in the Southeast Asia region
Interventions	Studies that reported about marine sites that are either being managed formally by the ‘national and local government’ or ‘non-governmental organisations’ or ‘people’s organisations’ in the Southeast Asia region, for environmental conservation	Studies without any intervention related to marine sites
Outcome measures	Poverty status in terms of economic or/and material living standards. We followed the definition according to the systematic map (Garside and Eales 2020 in press):	Studies that did not report poverty status in terms of the economic and material living status
	Economic living standards include measures of income, employment, employment opportunities, wealth/poverty, savings, payments, and loans	
	Material living standards include measures of access to and availability of food, fibre, fuel and basic infrastructure (electricity, water, telecommunications, and transportation); provision of shelter; and assets owned (e.g., television)	
Comparison	Any comparator which falls under one of the followings:	No comparator
	(i) A different site with no marine protection	
	(ii) The same site, before the marine protection was implemented	
	(iii) A different site, with another marine protection implementation	

At Stage 2, R1 will retrieve the full paper of the included abstracts. These papers will be divided among three pairs of reviewers (R1/R4, R2/R3, and R5/R6) who will conduct the full-text screening according to the eligibility criteria in Table 2.

Several studies that fulfil the PICO inclusion criteria will be shared with reviewers as learning tools before proceeding with the screenings. A list of studies rejected based on full-text assessment will be provided in an appendix of the full report, together with the reasons for exclusion. Consistency checking will be done at stages 1 and 2. At stage 1, approximately 10% of titles and abstracts will be double screened. Any discrepancies will be discussed and resolved between the two reviewers. Where both reviewers cannot reach agreement, the paper will move to the next stage. The reviewers will include a study whenever there is uncertainty regarding its relevance, e.g., when information in the abstract is deficient, unavailable, or missing. If the inter-rater agreement is lower than 80%, the double screening and consistency checking will be repeated for another 10% of the articles. Once at least 80% inter-rater agreement is reached, the remaining of the total number of the titles and abstracts will be divided into two for single screening at stage 1.

At stage 2, at least 10% of full texts will be screened by all the reviewers. Any discrepancies will be discussed and resolved by the whole reviewer group. If the inter-rater agreement is lower than 80%, the double screening and consistency checking will be repeated for another 10% of the articles. Once at least 80% inter-rater agreement is reached, the remaining of the total number of full texts will be divided so that each of the six reviewers can undertake single screening. Alongside percentage agreements, the kappa statistic will be used to check for inter-rater reliability [25].

## Study validity assessment

Eligible studies will be subjected to critical appraisal. The studies will be evaluated for methodological quality by using the Joanna Briggs Institute (JBI) critical appraisal checklist for cross sectional, case control, cohort, prevalence, and qualitative study types, given in Additional file 4. The JBI critical appraisal tools have been tested and extensively used for evidence syntheses in the field of healthcare. If there are any modifications that need to be made according to the difference between health (JBI designed the tools for health studies) and environmental studies, we will make these changes and clarify what these changes are in the final report. It is unlikely we will know exactly what these changes will be until we have tested a sample of eligible studies from our review.

## Data extraction strategy

Data from the included studies including the information on the study design, the measures of economic and material status, and the reported outcome will be extracted and summarised in a standardised evidence table. The extracted information will be based on the PICO elements, and the recorded outcomes will include study design, method used, date of data collection, population type (intervention), age category, ethnicity, description of intervention, comparator, and intervention outcomes (Additional file 5). The extracted information will be used to measure the effects of interventions on poverty in coastal communities in SEA.

When necessary, we will ask authors of relevant articles to supply data and/or further information. This will primarily be done when data/key information is either missing or insufficient.

## Potential effect modifiers and reasons for heterogeneity

Several potential effect modifiers that may contribute to heterogeneity in the outcome will be identified during the full-text screening and recorded for all the studies included in this review. A non-exhaustive list of potential effect modifiers is given as follows:

1. Weather and/or climatic conditions.
2. Socioeconomic status.
3. Type of fishing sectors (commercial, traditional, or recreational).
4. Likelihood of natural disaster (floods, typhoons, seismic/volcanic activity).
5. Sociocultural factors e.g., dominant religion, societal structure.
6. Size of communities (area and number of people).
7. Age-structure of communities.

## Data synthesis and presentation

A narrative synthesis of data from all studies included in the review will be generated to describe the strength and validity of the evidence base along with the study findings. Tables and figures will be produced to summarise these results. Where data are suitably comparable, meta-analysis will be considered. For this, the effect sizes will be standardised and weighted appropriately, for example, using the inverse of the variance.

If meta-analysis of effect sizes is possible, it will take the form of random-effects models. Full details of meta-analysis methods will be presented in the review report. Meta-regressions or subgroup analysis of categories of studies will also be performed where enough studies report common sources of heterogeneity. Publication bias and sensitivity analysis using critical appraisal categories will be carried out where possible. Overall effects will be presented visually in plots of mean effect sizes and variance in the systematic review.

## Supplementary Information

**Additional file 1: **Benchmark studies.

**Additional file 2: **ROSES checklist.

**Additional file 3: **Websites for additional grey literature searches.

**Additional file 4: **JBI critical appraisal checklist.

**Additional file 5: **Data extraction sheet.

## Data Availability

Not applicable.
