# Supplementary material for: What are the outcomes of marine site protection on poverty of coastal communities in Southeast Asia? A systematic review protocol
Source: Environ Evid. 2022 Feb 4;11:2. doi: 10.1186/s13750-022-00255-1 (PMC11378847; doi:10.1186/s13750-022-00255-1)
Supplement: Supplementary file 1 — Additional file 1: Benchmark studies. [file 13750_2022_255_MOESM1_ESM.docx]

**Additional file 1 - Estimating the comprehensiveness**

**List of benchmark articles**

| No | Title | Type |
| --- | --- | --- |
| 1 | Ballad, E. L., Morooka, Y., & Shinbo, T. (2017). Role of extension services with special reference to livelihood projects for supporting a community-based marine protected area in northern Luzon, Philippines. Asian Fisheries Science, 30(1), 1-16. https://www.asianfisheriessociety.org/publication/archivedetails.php?id=142,Philippines,18.268229,121.934321,Quantitative and Qualitative,Does not include mangroves (not clearly stated),N,Economic or livelihood incentives and alternatives,Economic living standards | Peer-reviewed published article |
| 2 | Rakhmanissazly, A., Permatasari, A. I., & Peranginangin, E. C. (2018, February). Edco-tourism; A Coastal Management Program to Improve Social Economics. In IOP Conference Series: Earth and Environmental Science (Vol. 116, No. 1, p. 012038). IOP Publishing. https://iopscience.iop.org/article/10.1088/1755-1315/116/1/012038,Indonesia,4.0601,98.2724,Quantitative and Qualitative,Includes mangroves,N,Economic or livelihood incentives and alternatives,Economic living standards | Peer-reviewed published article |
| 3 | Rola, A. C., Narvaez, T. A., Naguit, M. R. A., Elazegui, D. D., Brillo, B. B. C., Paunlagui, M. M., ... & Cervantes, C. P. (2018). Impact of the closed fishing season policy for sardines in Zamboanga Peninsula, Philippines. Marine Policy, 87, 40-50. https://doi.org/10.1016/j.marpol.2017.09.029,Philippines,6.9262,122.0789,Quantitative,Does not include mangroves (not clearly stated),N,Species management,Economic living standards | Peer-reviewed published article |
| 4 | Sangchumnong, A. (2018). Development of a sustainable tourist destination based on the creative economy: A case study of Klong Kone Mangrove Community, Thailand. Kasetsart Journal of Social Sciences. doi:10.1016/j.kjss.2018.02.002",https://doi.org/10.1016/j.kjss.2018.02.002,Thailand,13.3596,100.004,Quantitative and Qualitative,Includes mangroves,N,Economic or livelihood incentives and alternatives,Economic living standards | Peer-reviewed published article |
| 5 | Sari, R. P. (2013). What is the Relationship between Marine Protected Areas (MPAs) and Poverty in Indonesia? https://pdfs.semanticscholar.org/0263/5ed3c0d338aefb425f4728a08ab57fde0b51.pdf?_ga=2.76283410.603753536.1563362063-2083571992.1560513573,Indonesia, 2.3256,118.0053,Quantitative,Does not include mangroves (not clearly stated),Y,Site protection,Economic living standards | Peer-reviewed published article |
| 6 | Leisher, C., Carlton, V. A., Van Beukering, P., & Scherl, L. M. (2007). Nature's investment bank: how marine protected areas contributed to poverty reduction. https://www.researchgate.net/publication/288676649_Nature's_investment_bank_Marine_protected_areas_contribute_to_poverty_reduction,Indonesia,1.5232,124.6556,Quantitative and Qualitative,Does not include mangroves (not clearly stated),N,Site protection,Economic living standards | Grey literature organisational report |
| 7 | Pomeroy, R. S., Oracion, E. G., Pollnac, R. B., & Caballes, D. A. (2005). Perceived economic factors influencing the sustainability of integrated coastal management projects in the Philippines. Ocean & Coastal Management, 48(3-6), 360-377. http://dx.doi.org/10.1016/j.ocecoaman.2005.04.010,Philippines,9.6221,123.1352,Quantitative,Includes mangroves,N,Site protection,Economic living standards | Peer-reviewed published article |
| 8 | Triet, T. (2010). Combining biodiversity conservation with poverty alleviation-a case study in the Mekong Delta, Vietnam. Aquatic Ecosystem Health & Management, 13(1), 41-46. http://dx.doi.org/10.1080/14634980903566667,Vietnam,10.4394,104.5886,Quantitative and Qualitative,Does not include mangroves (not clearly stated),Y,Site protection,Economic living standards | Peer-reviewed published article |
| 9 | Fatimah, K., NurulHuda, M. S., & Salleh, N. H. M. (2012). Income Risk Vulnerability and Perception towards Conservation: A Community Level Analysis for Pulau Sibu-Tinggi MarinePark, Mersing. Journal of Tropical Marine Ecosystem, 2(1). http://journalarticle.ukm.my/5166/,Malaysia,2.298982,104.118741,Quantitative,Does not include mangroves (not clearly stated),N,Site protection,Economic living standards | Peer-reviewed published article |
| 10 | Salleh, N. H. M., Othman, R., Sarmidi, T., & Darawi, Z. (2011, July). A comparison of local community sustainability of livelihood: A case study in Redang and Tioman Islands, Malaysia. In 2011 IEEE International Summer Conference of Asia Pacific Business Innovation and Technology Management (pp. 144-148). https://ieeexplore.ieee.org/stamp/stamp.jsp?tp=&arnumber=5996310,Malaysia,2.7979,104.1687,Quantitative,Does not include mangroves (not clearly stated),N,Site protection,Economic living standards | Peer-reviewed published article |
| 11 | Eriksson, B., F. Johansson and M. Blicharska (2019). ""Socio-economic impacts of marine conservation efforts in three Indonesian fishing communities."" Marine Policy 103: 59-67. https://doi.org/10.1016/j.marpol.2019.02.007,Indonesia,-2.3256,118.0053,Qualitative,Does not include mangroves (not clearly stated),N,Site protection,Economic living standards | Peer-reviewed published article |
