# Supplementary material for: What are the outcomes of marine site protection on poverty of coastal communities in Southeast Asia? A systematic review protocol
Source: Environ Evid. 2022 Feb 4;11:2. doi: 10.1186/s13750-022-00255-1 (PMC11378847; doi:10.1186/s13750-022-00255-1)
Supplement: Supplementary file 2 — Additional file 2: ROSES checklist. [file 13750_2022_255_MOESM2_ESM.pdf]

Section / sub-section	Topic	Description	Further explanation	Checklist/Meta-data	Author response	Comments
Title	Title	review protocol, and must indicate if it is an	same or very similar to the	Meta-data	Yes	
Type of review	Type of review	systematic review, systematic review update,	amendments and updates [1]	Meta-data	systematic review	
Authors contacts	Authors contacts	email addresses for all authors must be		Checklist	Yes	
Abstract	Structured summary	include two sections 1) Background, the		Checklist	Yes	
Background	Background	context of what is already known. Protocol	conceptual model can be	Checklist	Yes	
Stakeholder engagement	Stakeholder engagement	throughout the review process (e.g. in		Checklist	Yes	
Objective of the review	Objective	questions (when applicable).	main question of the review.	Checklist	Yes	
	question components	elements e.g. population,	[3,4]	Meta-data	Yes	
Methods						
Searches	Search strategy	including: database names accessed,	strategy testing should be	Checklist	Yes	
	Search string	state the platform for which the string is		Meta-data	Yes	
	bibliographic databases	database searches.		Meta-data	Yes	
	literature	websites searches and web-based search		Meta-data	Yes	
	Bibliographic databases	to be searched.		Meta-data	Yes	
	engines	engines to be searched.		Meta-data	Yes	
	Organisational websites	to be searched.		Meta-data	Yes	
	comprehensiveness of	comprehensiveness of the search strategy was		Checklist	Yes	
	Search update	during the conduct of the review.	good practice if original	Checklist	n/a	
study inclusion criteria	Screening strategy	articles/studies for relevance/eligibility.		Checklist	Yes	
	Consistency checking	consistency of decisions including the levels at		Checklist	Yes	
	Inclusion criteria	relevance of identified articles/studies. These		Checklist	Yes	
	Reasons for exclusion	excluded at full text with reasons for exclusion.		Checklist	Yes	
Critical appraisal	Critical appraisal strategy	critical appraisal of study validity (including		Checklist	Yes	
	synthesis	appraisal will be used in synthesis.		Checklist	Yes	
	Consistency checking	of study validity will be tested.		Checklist	Yes	
Data extraction	coding strategy	and coding for studies (potentially providing		Checklist	Yes	
	Data extraction strategy	qualitative and/or quantitative study findings		Checklist	Yes	
	data	confirming missing or unclear information or		Checklist	Yes	
	Consistency checking	data/data extraction process will be tested.		Checklist	Yes	

modifiers/reasons for	modifiers/reasons for	modifiers /reasons for heterogeneity that will	exhaustive but a short list of	Checklist	Yes
presentation	Type of synthesis	the systematic review (narrative only, narrative		Meta-data	NA
	strategy	synthesising the evidence base in the form of	studies based on the direction	Checklist	Yes
	strategy	synthesis, describe planned methods for	data	Checklist	Yes
	strategy	qualitative data and justify your methodological	data	Checklist	Yes
	strategies	synthesising data or combining qualitative and	data	Checklist	Yes
	publication bias	possible influence of publication bias on the	may be done using diagnostic	Checklist	systematic review from a systematic map
	identification strategy	and/or prioritise key knowledge gaps	Optional	Checklist	n/a
	procedural	have also authored articles to be considered	articles to be considered within	Checklist	Yes
Declarations	Competing interests	competing interests that the review authors		Checklist	Yes

References

[1] Bayliss, H.R., Haddaway, N.R., Eales, J., Frampton, G.K. and James, K.L., 2016. Updating and amending systematic reviews and systematic maps in enviro

[2] Haddaway, N.R., Kohl, C., da Silva, N.R., Schiemann, J., Spök, A., Stewart, R., Sweet, J.B. and Wilhelm, R., 2017. A framework for stakeholder engagement

[3] Collaboration for Environmental Evidence. 2018. Guidelines and Standards for Evidence synthesis in Environmental Management. Version 5.0. www.env

[4] Leeds Institute of Health Sciences. https://medhealth.leeds.ac.uk/info/639/information_specialists/1500/search_concept_tools. Accessed 12/11/2017.
