# Supplementary material for: What are the outcomes of marine site protection on poverty of coastal communities in Southeast Asia? A systematic review protocol
Source: Environ Evid. 2022 Feb 4;11:2. doi: 10.1186/s13750-022-00255-1 (PMC11378847; doi:10.1186/s13750-022-00255-1)
Supplement: Supplementary file 3 — Additional file 3: Websites for additional grey literature searches. [file 13750_2022_255_MOESM3_ESM.docx]

Additional file 3: Additional grey literature searches websites

| No. | Website | URL |
| --- | --- | --- |
| 1. | Philippine Institute for Development Studies | https://pids.gov.ph/ |
| 2. | Palawan Council for Sustainable Development | https://www.pcsd.gov.ph/aboutpcsd/index.htm |
| 3. | Universiti Sains Malaysia | <http://www.usm.my/index.php/en/>  directories/websites/research |
| 4. | PROQUEST | https://search.proquest.com/pqdtglobal/  index?_ga=2.73652754.1011994634.1530626827-1292196482.1530626827 |
| 5. | University of Hasanuddin, Makassar | https://unhas.ac.id/en/page/Library |
